# A c*linical reading* on “World Allergy Organization-McMaster University Guidelines for Allergic Disease Prevention (GLAD-P): Probiotics”

**DOI:** 10.1186/s40413-016-0101-8

**Published:** 2016-03-10

**Authors:** Giampaolo Ricci, Francesca Cipriani, Carlos A. Cuello-Garcia, Jan L. Brożek, Alessandro Fiocchi, Ruby Pawankar, Juan José Yepes-Nuñes, Luigi Terraciano, Shreyas Gandhi, Arnav Agarwal, Yuan Zhang, Holger J. Schünemann

**Affiliations:** Department of Clinical Epidemiology & Biostatistics, McMaster University, Hamilton, Ontario Canada; Tecnologico de Monterrey School of Medicine, Monterrey, Mexico; Department of Medicine, McMaster University, Hamilton, Ontario Canada; Pediatric Hospital Bambino Gesù, Rome, Vatican City, Italy; Department of Pediatrics, Nippon Medical School, Tokyo, Japan; Department of Child and Maternal Medicine, University of Milan Medical School at the Melloni Hospital, Milan, Italy; Faculty of Medicine, University of Toronto, Toronto, Ontario Canada; Pediatric Unit, Department of Medical and Surgical Sciences, University of Bologna, S. Orsola-Malpighi Hospital, Pad 16, via Massarenti 11, 40138 Bologna, Italy

**Keywords:** Allergy, Atopy, Eczema, Lactobacillus, Prevention, Probiotic, Supplementation

## To the Editor

Since the immunomodulatory properties of probiotics have been described, the effect of probiotic supplementation has been investigated in several trials and it has been also proposed as a preventive intervention for the development of allergic diseases. Recently two important evidence-based recommendations about the use of probiotics in the prevention of allergy were published [1, 2] with conflicting conclusions, in particular the most recent guideline [1] seems to be partially in contradiction with the previous statements about prevention of eczema. For these reasons, we tried to analyze the evidences leading to these recommendations [1] to highlight the aspects that can be more directly related or correlated with clinical practice. This *clinical reading* was addressed to offer some reflections about the methods used to formulate such recommendations, and the possibility to adopt in the clinical practice the proposed conclusions.

We tried to retraces the path proposed by the Authors to analyse three important questions about the efficacy of probiotics in preventing allergic diseases if administrated to pregnant women (first question), to breastfeeding mothers (second question) and in healthy infants (third question).

These questions have been investigated by using the Grading of Recommendations Assessment, Development and Evaluation (GRADE) approach [3], to perform a systematic review of randomized controlled trials and formulate recommendations. In summary, the GRADE method aims to define a rigorous and explicit method for the production of clinical recommendations. According to this method, the knowledge of advantages and disadvantages, benefits and risks of an intervention is necessary to make decisions in the health field. The GRADE approach also provides a three-phases decisional framework: a) formulation of a clinical question, with the choice and the formal evaluation of its related outcomes, and systematic evaluation of the scientific literature and the quality of the evidence; b) evaluation of the benefits and risks associated to the intervention, taking also in consideration its feasibility, the necessary resources and the patients’ preferences; c) formal definition of the strength of the recommendation.

From a methodological point of view, it should be noted that the recommendations are directed to patients, clinicians and other health care professionals with different objectives, as clearly explained in the guidelines introduction. Each recommendation can have different levels of strength: strong recommendation indicates that patients would like to receive the intervention and that clinicians should actuate it. Conditional or weak recommendation indicates that the majority of patients would like to receive the intervention, but many other not, as well as they hope that clinicians would recognize that different choices may be appropriate for different patients, by taking advantage of decision-making tools to help patients to make consistent choices.

In the results section of the guidelines we can read that “Currently available evidence does not indicate that probiotic supplementation reduces the risk of developing allergy in children. However, considering all critical outcomes in this context, the World Allergy Organization (WAO) guideline panel determined that there is a likely net benefit from using probiotics resulting primarily from prevention of eczema” [1].

In the first sentence, the guideline panel confirmed the absence of evidence of effectiveness in the use of probiotics in the primary prevention of the common allergic diseases (asthma, food allergy, rhinitis). The second sentence deserves a careful review, because it seems to contradict the meaning of the first sentence and the position of the other guidelines, including the recent European Academy of Allergy and Clinical Immunology (EAACI) guideline [2].

In order to comment this phrase, we started from the description of the evidences (GRADE) in Appendix 2 - Question 1: “Should probiotics vs. no probiotics be used in pregnant women?” In this appendix we can found declared how many studies addressing this question have been selected for each of the clinical objectives: 15 randomized trials were considered for the prevention of eczema.

The comment of this question can be also reliable if it is applied to the other two questions above, as approximately the same studies have been selected for critical review.

In this regard, we carefully examined the individual trials to better analyze and understand the results of the question. The most important clinical features that supported our considerations are reported in Table 1 and Table 2.

**Table 1:** Characteristics of the 15 trials included in the meta-analysis and addressing the guideline’s Question 1: “Should probiotics vs. no probiotics be used in pregnant women?”.

References	Population at risk of atopy	N° patients	Probiotic	Duration of ante-partum therapy	Duration of therapy in breastfed infants	Duration of therapy in infants fed with formulas
Kalliomaki et al. 2001 [4]	yes	159	*Lactobacillus rhamnosus GG (ATCC 55103)*	2-4 weeks	6 months	6 months
Rautava et al. 2002 [5]^a^	yes	62	*Lactobacillus rhamnosus GG (ATCC 53103)*	4 weeks	3 months	3 months
Abrahamsson et al. 2007 [6]	yes	232	*Lactobacillus reuteri (ATCC55730)*	4 weeks	12 months	12 months
Kukkonen et al. 2007 [7]	yes	1223	*Lactobacillus rhamnosus GG (ATCC 55103)*, *Lactobacillus rhamnosus LC705 (DSM7061)*, *Bifidobacterium breve Bb99 (DSM13692)*, *Propionibacterium freudenreichiii ssp shermanii JS (DSM7076*)	2-4 weeks	6 months	6 months
Huurre et al. 2008 [8]	yes	171	*Lactobacillus rhamnosus GG (ATCC 55103)* *Bifidobacterium lactis Bb12*	6 months	end of breastfeeding	-
Marschan et al. 2008 [9]^b^	yes	98	*Lactobacillus rhamnosus GG (ATCC 55103), * *Lactobacillus rhamnosus LC705 (DSM7061)*, *Bifidobacterium breve Bb99 (DSM13692), * *Propionibacterium freudenreichiii ssp shermanii JS (DSM7076*)	2-4 weeks	6 months	6 months
Wickens et al. 2008 [10]	yes	512	*Lactobacillus rhamnosus HN001, * *Bifidobacterium animalis subspec lactis HN019*	5 weeks	6 months	24 months
Kopp et al. 2008 [11]	yes	105	*Lactobacillus rhamnosus GG ( ATCC 53103)*	4-6 weeks	6 months	6 months
Niers et al. 2009 [12]	yes	156	*Bifidobacterium bifidum W23*, *Bifidobacterium lactis W52* *Lactococcus lactis W58*	6 weeks		12 months
Dotterud et al. 2010 [13]	no	415	*Lactobacillus rhamnosus GG*, *Bifidobacterium animalis subsp.Bb-12*, *Lactobacillus acidophilus La-5*	4 weeks	3 months	
Kim et al. 2010 [14]	yes	112	*Bifidobacterium bifidum BGN4*, *Bifidobacterium lactis AD011, * *Lactobacillus acidophilus AD031*	8 weeks	3 months	4-6 months
Boyle et al. 2011 [15]	yes	250	*Lactobacillus rhamnosus GG ( ATCC 53103)*	4 weeks		
Ou et al. 2012 [16]	Yes (maternal hystory)	191	*Lactobacillus rhamnosus GG ( ATCC 53103)*	4 months	6 months	6 months
Allen et al. 2012 [17]	the most of partecipants	454	*Lactobacillus salivarius CUL61, * *Lactobacillus paracasei CUL08*, *Bifidobacteriums animalis ssp. lactis CUL34, * *Bifidobacterium bifidum CUL20*	4 weeks	6 months	6 months
Rautava et al. 2012 [18]	yes	241	*Lactobacillus rhamnosus LP + Bifidobacterium longum (ATCC BAA-999)* *or* *Lactobacillum paracasei ST11 + Bifidobacterium longum (ATCC BAA-999 )*	2 months	2 months	

^a^Analysis of a subgroup of patients previous analyzed by Kalliomaki et al. [4]

^b^Analysis of a subgroup of patients previous analyzed by Kukkonen et al. [7]

**Table 2:** Main clinical characteristics related to the diagnosis of eczema in 15 trials included in the meta-analysis for the guideline’s Question 1: “Should probiotics vs. no probiotics be used in pregnant women?”.

References	Duration of follow up (months)	Diagnostic criteria for eczema	Patients evaluable at the end of follow-up period	Persistence of eczema			Allergic sensitization	
				PROBIOTIC	PLACEBO	*P value*	%	*P value*
Kalliomaki et al. 2001 [4]	24	Harrigan 1999	64/77 68/82 (Pl)	15/64 23 %	31/68 46 %	0.008	(sIgE) 27 % vs 25 % (Pl) (SPT) 18 % vs 14 % (Pl)	NS NS
Rautava et al. 2002 [5]^a^	24	Harrigan 1999	32 30 (Pl)	4/27 15 %	14/30 47 %	0.0098	(sIgE) 28 % vs 37 % (Pl) (SPT) 23 % vs 21 % (Pl)	NS NS
Abrahamsson et al. 2007 [6]	24	H-R	95/117 93/115(Pl)	36 % IgE-Eczema 8 %	34 % IgE-Eczema 20 %	NS 0.02	(sIgE) 37 % vs 48 % (SPT) 18 % vs 29 %	NS NS
Kukkonen et al. 2007 [7]	24	UK-WP	461/610 464/613 (Pl)	26 % IgE-Eczema 12.4 %	32.3 % IgE-Eczema 17.7 %	0.035 0.025	(sIgE e/o SPT) 28 % vs 31.2 (Pl)	NS
Huurre et al. 2008 [8]	12	H-R	72 68 (Pl)	9.7 %	17.6 %	NS	29 % vs 31 % (Pl) Subgroup with maternal sensitization. 26 % vs 50 % (Pl)	NS 0.023
Marschan et al. 2008 [9]^b^	24	UK-WP	52 46 (Pl)	31 %	39 %		(sIgE) 35 % vs 26 % (Pl)	
Wickens et al. 2008 [10]	24	UK-WP	144/157 152/158 150/159 (Pl)	*Lrha* 14.8 % *Bl* 24.2 % IgE-Eczema *Lrha* 9.9 % *Bl* 12.8 %	26.8 % 18.5 %	0.03 NS 0.04 NS	(SPT) * Lrha* 21.3 % e Bl 23.5 % vs 28.8 % (Pl)	NS
Kopp et al. 2008 [11]	24	UK-WP	50/54 44/51 (Pl)	28 %	27.3 %	NS	(sIgE to inhalants) 8 % vs 11.3 % (Pl)	NS
Niers et al. 2009 [12]	24	H-R	50/78 48/78 (Pl)	(Questionnaire) 3 months 12 % 24 months 54 % (Clinical) 3 months 6 % IgE-eczema 20 %	(Questionnaire) 3 months 29 % 24 months 68.7 % (Clinical) 3 months 21 % IgE-eczema 16.6 %	0.035 0.05 0.021 NS	(sIgE o SPT) 20 % vs 14.6 % (Pl)	NS
Dotterud et al. 2010 [13]	24	UK-WP	138/211 140/204 (Pl)	21 % IgE-Eczema 6.9 % Non IgE-Eczema 13 %	34.3 % IgE-Eczema 7.5 % Non IgE-Eczema 25.6 %	0.022 NS 0.009	(IgEs o SPT) 15.3 % vs 11.3 % (Pl)	NS
Kim et al. 2010 [14]	12	H -R	33/57 35/55 (Pl)	36.4 % IgE-eczema 9.7 %	62.9 % IgE-eczema 20.7 %	0.029 NS	(sIgE) 38.7 % vs 51.7 % (Pl)	NS
Boyle et al. 2011 [15]	12	UK-WP	109/125 103/125 (Pl)	32 %	42 %	NS	(SPT) 33 % vs 33 % (Pl)	NS
Ou et al. 2012 [16]	36		65/95 63/96 (Pl)	6 months 3.3 % 18 months 25 % 36 months 24.6 %	6 months 23.6 % 18 months 17.7 % 36 months 25 %	NS NS NS	Allergic symptoms 47.7 % vs 46.9 % (Pl)	NS
Allen et al. 2012 [17]	24	Clinical	187/220 172/234 (Pl)	34.1 % IgE-eczema 5.3 %	32.4 % IgE-eczema 12.1 %	NS 0.024	10.5 % vs 18.5 %	0.036
Rautava et al. 2012 [18]	24	H-R	73/81 (Lrha) 70/82 (Lpar) 62/78 (Pl)	29 % (10 %)* 29 % (6 %)*	71 % (26 %)*	0.001 0.001	(SPT) 22 % 26 % 26 % (Pl)	NS

H-R: Hanifin and Rajka; UK-WP: UK Working Party; Harrigan 1999: Harrigan’s criteria [28]; *Lrha*: *Lactobacillus rhamnosus; Bl: Bifidobacterium lactis; Lpar*: *Lactobacillus paracasei*; Pl: Placebo; SPT: skin prick test; sIgE: specific IgE

^a^Analysis of a subgroup of patients previous analyzed by Kalliomaki et al. [4]

^b^Analysis of a subgroup of patients previous analyzed by Kukkonen et al. [7]

*Persistence of eczema *P* < 0,003

A preliminary observation about the 15 trials above [4–18] is that they are all randomized against placebo, they were published between 2001 and 2012, that is a rather brief period to be well compared. It should be mentioned that the study of Rautava et al. (2002) [5] analyzes a subset of patients previous described by Kalliomaki et al. (2001) [4], as well as Marschan et al. (2008) [9] analyzed a subgroup of patients described by Kukkonen et al. (2007) [7].

Almost all studies identified patients with family history of atopy as targets for treating, indicating that children at high risk of atopy should be the potential beneficiaries of a preventive intervention with probiotic supplementation.

The total number of subjects included in the trials is relevant, but the type of probiotic or association of probiotics used is widely different among the studies. As the Authors underlined, we have to face a "heterogeneity of the interventions and limitations in reporting of original studies”, so it was not possible to analyze neither the effects in each group separately nor the effects of individual probiotic species.

This statement is clearly expressed in the guideline, but it deserves some particular considerations. The term *probiotic* currently includes bacteria associated with beneficial effects; according to the World Health Organization definition [19], the term *probiotics* refers to “live micro-organisms which, when administered in adequate amounts, confer a health benefit on the host". Probiotics may act by different mechanisms, as well as the Authors have pointed out, that means it is important to consider also if the action is the same or if there are differences of action between the different probiotics used in the different trials. Since a wide literature is available on probiotics, we can take for example the data related to *Lactobacillus rhamnosus,* the most used probiotic species.

A recent trial examined 100 strains of *Lactobacillus rhamnosus* isolated from different food sources and habitats from the human body (mouth, bowel, vagina) [20] by carrying out a genomics and functional comparative analysis. A wide spectrum of phenotypes with different functional properties, which have been grouped by the Authors according to the two most common geno-phenotypes (A and B). The geno-phenotype A seems to be related permanently to a greater ability to adapt to nutrient-rich foods such as dairy products, but also to the loss of some biological functions involved in the antimicrobial activity, in the resistance and ability to adapt to different habitats. The geno-phenotype B shows a greater ability to adapt to different habitats and resources with different nutrients and different effects on the host.

A recent study analyzed the relationship between dental caries and *Lactobacillus rhamnosus,* showing a likely causal role of two strains: *Lactobacillus rhamnosus* LRHMDP2 and LRHMDP were isolated in the dental pulp, furthermore the analysis of their genes showed differences with the probiotic *Lactobacillus rhamnosus* GG (ATCC 53103) which does not seem implicated in the pathogenesis of dental caries [21].

Also a comparison between Lactobacillus strains isolated from dairy products market of *Lactobacillus rhamnosus* GG (ATCC53103), *Lactobacillus rhamnosus* LRV, *Lactobacillus rhamnosus* LRI, *Lactobacillus casei* BL23 (ATCC393), *Lactobacillus casei* LCY, and *Lactobacillus casei* LCA showed different genomic profiles, as well as different functional capabilities in glucose metabolism and in the ability to adhere to intestinal mucus [22]. As the Authors also point out, we believe that this evidence is a critical point for the definition of the quality of the evidence, described as very low.

In the selected studies, different strains have been used in various combinations: it is necessary to consider different mechanisms of action and possible interactions in the bowel, in addition to the different effects of different combinations of micro-organisms. The species *Lactobacillus rhamnosus* GG (ATCC 55103) is the most widely studied probiotic, both alone and in combination, but the data only from studies that use this type of probiotic would be insufficient.

The duration of treatment in the late pregnancy is fairly homogeneous across all studies, and it varies from 2 to 6 weeks before the expected date of birth.

Among the 15 selected studies, we considered the main clinical features associated with the diagnosis of eczema useful to analyze the question 1 of the guideline and we reported them in Table 2. The predominant duration of follow-up was 24 weeks, a sufficient period to evaluate the appearance of atopic dermatitis, since in most cases it begins within 24 months of life. The criteria used for the diagnosis of atopic eczema are the most widely recognized in the literature: Hanifin and Rajka criteria [23] and the UK-Working Party’s criteria [24]. The number of patients with a complete follow-up usually exceed the 80 % of the enrolled patients, so the quality of the available data seems to be very good for the statistical analysis.

It is more difficult to evaluate the results on the occurrence of eczema with its two clinical phenotypes: the IgE-associated and non IgE-associated, which differ both in the clinical features and in the evolution toward other allergic diseases. IgE-associated forms are more prone to the evolution in allergic rhinitis and asthma, especially if there is also a clear sensitization to inhalant allergens. The data shown in Table 2 at the end of follow-up period include 13 trials, excluding the two trials above mentioned [5, 9] that analyzed subsets of patients of broader studies [4, 7]. Among these, 6 studies [4, 7, 10, 13, 14, 18] showed a significant reduction in the rate of occurrence of eczema in the group treated with probiotics if compared with placebo group, while the other 8 studies [6, 8, 10–12, 15–17] didn’t show significant differences between the two groups. The study of Wickens et al. [10] is considered twice as the group receiving *Lactobacillus rhamnosus* HN001 was statistically significant while that one treated with *Bifidobacterium animalis lactis subspec* HN019 was not significant.

In 7 of these trials, the analysis can be restricted to the appearance of IgE-associated eczema: 4 studies showed a statistical significance [6, 7, 10, 17], while 4 studies [10, 12–14] didn’t show significant differences (the study of Wickens et al. [10] was considered twice).

Finally, the analysis of the presence of allergic sensitization assessed by specific IgE and/or skin prick test provided significant results only in two cases, while in 13 others it was not statistically significant.

Another important issue discussed in this guideline is the detection of adverse effects. The analysis took into consideration also an extensive review of a study carried out by the Health Assessment Technology [25], which did not report significant adverse effects and in any case, it did not describe an increased risk, despite some limitations indicated in the study. Lastly, the adverse events reported in these 15 trials are mild and short-term events, without substantial clinical differences between the groups receiving probiotics and those receiving placebo.

## Conclusions

This guideline indicates the lack of clear evidence of efficacy of probiotic supplementation in the primary prevention of allergies. In the recommendation 1, the WAO guideline panel suggests "using probiotics in pregnant women at high risk for allergy in their children, because considering all critical outcomes, there is a net benefit resulting primarily from prevention of eczema (conditional recommendation, very low quality evidence)”; in the case of eczema this recommendation is also provided regarding the other two clinical questions.

The low number of reported adverse effects confirms a high safety profile. The "net benefit" highlighted in the prevention of eczema appears to be overly optimistic when considering the wide heterogeneity of interventions and the inconsistence of the results in the statistical analysis. However, the GRADE system takes into account aspects that are helpful to "support each person in reaching a management decision consistent with his/her values and preferences" [3]. Nevertheless, although we cannot exclude the possibility of using probiotics in special situations, such as an important family history of atopic eczema, the extension and the generalization of their use does not seem to be sufficiently supported by scientific evidence.

Eczema and allergic diseases represent a complex puzzle in which many factors interact. It is often difficult to separate the various factors involved, also because some of their effects may develop after a long time and are difficult to demonstrate. The guideline provides an important scientific synthesis that allows us to highlight the limitations of certain studies, but at the same time to get good points for further research. The time to suggest that preventive approach in a generalized way has not yet come, but these findings may be the starting point to search for more solid evidence and for new trials.

### Author’s response to “A c*linical reading* on “World Allergy Organization-McMaster University Guidelines for Allergic Disease Prevention (GLAD-P): Probiotics”

Cuello-Garcia CA, Brożek JL, Fiocchi A, Pawankar R, Yepes-Nuñez JJ, Terracciano L, Gandhi S, Agarwal A, Zhang Y, Schünemann HJ

We would like to thank Drs. Ricci and Cipriani for their insightful comments on the World Allergy Organization (WAO) guideline for the prevention of allergies with probiotics. In their letter, Drs. Ricci and Cipriani raised three concerns calling for additional clarification:

- the discrepancy in the conclusions between the WAO [1] and the European Academy of Allergy and Clinical Immunology (EAACI) guidelines [2] regarding the use of probiotics;
- the possibility to generalize recommendations to use probiotics when children are not at high risk of developing allergy; and
- a possibly “overly optimistic” assessment of benefits in the face of heterogeneity of interventions and inconsistency of the results in the analysis.

The main focus of the EAACI guidelines was on prevention of food allergy, whereas the WAO guidelines focused on a broader question whether or not probiotics should be used in the context of prevention of any allergy in otherwise healthy infants. The WAO and EAACI experts’ conclusion about the paucity of evidence was the same – both found not enough published evidence to confirm or reject an effect of probiotic supplementation on development of *food allergy* in infants. However, in order to provide advice whether or not to use probiotics for the prevention of allergy, the WAO guideline panel considered all outcomes important to patients and their caregivers in this context. Those included development of food allergy but also other outcomes deemed by the WAO panel to be important: development of wheezing and/or asthma, allergic rhinitis, eczema, urticaria and anaphylaxis, nutrition status, infection with probiotic bacteria and other adverse effects.

WAO advice to use probiotics in pregnant women, breastfeeding mothers and infants at high risk of developing an allergy was explicitly based on the likely lower risk of developing eczema. The recommendation stated, “….there is a net benefit resulting primarily from prevention of eczema” and that “…there is lack of evidence that probiotics prevent any other allergy”. This was a conditional recommendation meaning that clinicians should recognize that different choices would be appropriate for different individuals, often depending on their values and preferences. The WAO guideline panel, again explicitly, informed that the evidence supporting this advice was of very low quality, meaning that the panel was very uncertain how probiotic supplementation would actually affect all outcomes of interest. This implies that any further research – if done – is very likely to change both the strength and even the direction of those recommendations.

Drs. Ricci and Cipriani also expressed a concern about the possibility of broadening the recommendations to use probiotic supplementation to situations when infants would be at low risk of developing allergies. The WAO guideline panel was again very explicit suggesting using probiotics in infants at high risk. Usually, one can extrapolate from the evidence in high-risk populations to lower risk groups with additional uncertainty about the expected effects introduced by the lack of direct evidence. In the case of WAO recommendations, the guideline panel thought that this additional extrapolation from already indirect evidence would make any information too unreliable to support an informed advice for low risk situations.

The last comment is in relation to a possibly “overly optimistic” assessment of net benefits from probiotic supplementation in the face of heterogeneity of probiotics used in the studies and inconsistency of the results in the analysis. Dr. Szajewska and colleagues have previously expressed similar concerns whether the effect of probiotics on preventing eczema is a class effect or if one probiotic might have better efficacy than others might [26]. We share these concerns and agree that a conclusion that all probiotics are equal would be premature. However, the available data from randomized trials do not exclude the possibility of either a class effect or a true difference among the probiotic strains in their effects in prevention of eczema. This very inconsistency in the results among studies and other limitations resulted in previously mentioned very low confidence in the effects of probiotics [27].

We agree with Drs. Ricci and Cipriani that more research evidence is needed. Until it becomes available, parents and clinicians will need to make decisions under uncertainty about the true balance of benefits and downsides of using probiotic supplementation in pregnant women, breastfeeding mothers and infants with the intention to prevent allergies.
